# Takotsubo Cardiomyopathy Following an Emergent Midline Exploratory Laparotomy: A Case Report

**DOI:** 10.7759/cureus.97655

**Published:** 2025-11-24

**Authors:** Diya' A Abu Ghazal, Ahmed A Khalil, Jawdat Abu Ghazal, Mohammad A Abu Ghazal, Osama Alsayed

**Affiliations:** 1 Faculty of Medicine, Cairo University, Cairo, EGY; 2 Department of Cardiology, Kasr Al-Ainy, Cairo University Hospitals, Cairo, EGY

**Keywords:** acute coronary syndrome, laparotomy, left ventricular dysfunction, stress-induced cardiomyopathy, takotsubo cardiomyopathy

## Abstract

Takotsubo cardiomyopathy (TCM), also known as stress-induced cardiomyopathy, is a transient left ventricular dysfunction often precipitated by acute emotional or physical stress. We present a case of a 45-year-old male patient who developed Takotsubo cardiomyopathy subsequent to an emergent laparotomy. Postoperative chest pain and ST-segment elevation on electrocardiogram (ECG) raised the concern for acute coronary syndrome; however, coronary angiography revealed no obstructive lesions. This case emphasizes the importance of including Takotsubo cardiomyopathy in the differential diagnosis of perioperative cardiac events, even in atypical patient profiles.

## Introduction

Takotsubo cardiomyopathy, also known as stress-induced cardiomyopathy, represents transient systolic and diastolic left ventricular dysfunction. In 1990, Takotsubo cardiomyopathy was first reported in Japan. It took its name from a traditional Japanese octopus trap, which resembles the left ventricular apical ballooning morphology characteristic of this condition [1]. Clinically, Takotsubo cardiomyopathy often mimics acute coronary syndrome, making early delineation of coronary anatomy important. Depending on the clinical circumstances, this can be achieved using invasive coronary angiography or multi-slice CT (MSCT) [2]. Takotsubo cardiomyopathy is diagnosed using the modified Mayo Clinic criteria, including transient left ventricular wall motion abnormalities, absence of coronary obstruction, ECG changes, mild biomarker elevation, and exclusion of myocarditis or pheochromocytoma [3,4]. Although the exact pathophysiology remains unclear, most triggers of Takotsubo cardiomyopathy share a common mechanism: a state of excessive catecholamine release, which can be precipitated by emotional or physical stress [5]. In this paper, we present a case of Takotsubo cardiomyopathy precipitated by an emergent surgical intervention for a perforated duodenum.

## Case presentation

A 45-year-old male patient presented to the emergency department with acute epigastric pain of one day's duration that progressed to generalized abdominal pain. The condition was associated with abdominal distention and bilious vomiting.

The patient is a chronic heavy smoker, cannabis user, and Tramadol abuser, with a history of a recent ischemic stroke affecting the right basal ganglia three months ago, with residual insult in the form of residual left-sided weakness and rightward deviation of the mouth. The patient denied any increases in stress or emotional state prior to presentation.

On initial presentation, the patient was hypotensive (blood pressure (BP): 90/65 mmHg), tachycardiac (heart rate (HR): 130 beats per minute), tachypneic (respiratory rate (RR): 28 breaths per minute), maintained normal oxygen saturation (SpO_2_) on room air (98%), and febrile with a temperature of 38.5°C. Abdominal examination was remarkable for a rigid abdomen with diffuse abdominal tenderness and rebound tenderness. Both laboratory findings and blood gas analysis were significant for a calcium of 8.1 mg/dL, a creatinine of 1.54 mg/dL, a blood urea nitrogen of 72 mg/dL, a white blood cell count of 13.1 K/mm³, and a pH of 7.31 with a bicarbonate of 20 mmol/L (Table 1).

**Table 1:** Patient's laboratory values.

Investigations	Value	Normal Range and Units
Sodium	140.2	135–145 mmol/L
Potassium	4.5	3.5–5.3 mmol/L
Calcium	8.1 (Low)	8.4–10.2 mg/dL
Alanine aminotransferase (ALT)	54	10–56 U/L
Bilirubin – total	1.02	0.1–1.0 mg/dL
Bilirubin – direct	0.19	0.0–0.3 mg/dL
International normalized ratio (INR)	1.08	0.8–1.2
Creatinine	1.54 (High)	0.6–1.3 mg/dL
Blood urea nitrogen	72 (High)	7–22 mg/dL
Glucose	99	70–110 mg/dL
White blood cell count	13.1 (High)	4.1–9.3 K/mm³
Red Blood cell count	4.75	3.66–5.56 M/mm³
Hemoglobin	15.4	13.8–17.2 gm/dL
Hematocrit	48	40.6–51.8%
Platelet count	260	150–450 K/mm³
pH	7.31 (Low)	7.35–7.45
Carbon dioxide	44	35–45 mmHg
Bicarbonate	20 (Low)	22–28 mmol/L

**Figure 1:**
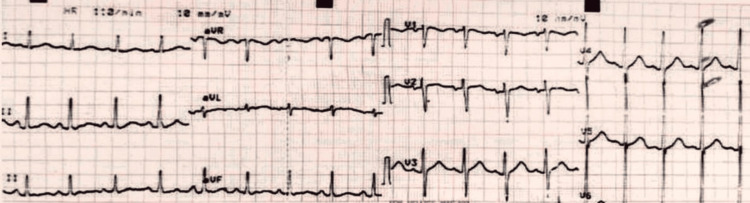
Patient's preoperative electrocardiogram shows sinus tachycardia with no other specific changes.

Further investigations were performed, including a pelvic-abdominal ultrasound, which showed moderate turbid free fluid with air foci under the diaphragm in the right and left subphrenic and subhepatic regions. Preoperative electrocardiogram (ECG) showed sinus tachycardia with no other specific changes (Figure 1). Transthoracic echocardiography, dating to his last hospitalization with stroke, showed normal left ventricular dimensions and function.

An open midline exploratory incision was done, revealing a perforated duodenal ulcer measuring 0.5 x 0.5 cm, for which Graham's (omental) patch was done.

**Figure 2:**
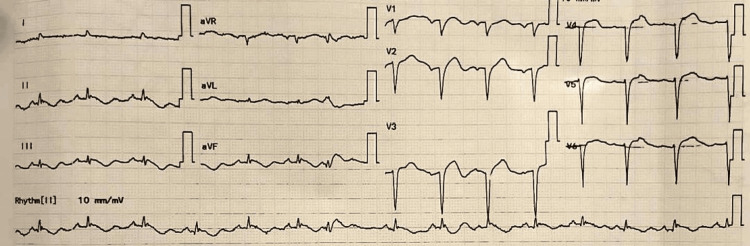
Patient's postoperative electrocardiogram shows new ST-segment elevation and Q waves in the anterior precordial leads (V1 to V4).

Postoperatively, the patient was extubated and monitored in the surgical intensive care unit (ICU). Ten hours later, the patient developed compressive retrosternal chest pain associated with profuse diaphoresis. At that time, the patient was hemodynamically stable, with a BP of 105/70 mmHg, HR of 99 beats per minute, RR of 23 breaths per minute, temperature of 37.5°C, and SpO_2_ of 98% on room air. An immediate electrocardiogram was done, which showed sinus tachycardia along with new onset upward convex ST-segment elevation and Q waves in the anterior precordial leads (V1 to V4) (Figure 2).

Immediate cardiac enzymes were also drawn, showing an elevation of high-sensitivity cardiac troponin to 8.8 (upper reference limit is 0.05), elevated creatine kinase (CK) up to 1104 IU/L (normal range 0-170 U/L), and elevated creatine kinase-myocardial band (CK-MB) up to 113.8 (normal range < 25 U/L), which indicated myocardial injury.

The transthoracic echocardiography revealed mildly reduced contractility, with an estimated ejection fraction (EF) of 45% and an akinetic apex and adjacent segments (Videos 1-3).

**Video 1:** Transthoracic echocardiography apical four-chamber view showing akinetic apex and adjacent apical segments.

**Video 2:** Transthoracic echocardiography apical three-chamber view showing akinetic apex and adjacent apical segments.

**Video 3:** Transthoracic echocardiography apical two-chamber view showing akinetic apex.

The differential diagnoses in this phase were possible myocardial infarction (whether type 1 or type 2) or Takotsubo cardiomyopathy (see Videos 1-3). The patient was immediately transferred to the catheterization laboratory for an emergency coronary angiogram. Coronary angiography was performed via radial access, revealing no obstructive coronary lesions (Videos 4, 5).

**Video 4:** Different angiographic views of the left coronary system showing no significant obstructive coronary artery disease.

**Video 5:** Angiographic views of the right coronary artery showing no significant obstructive coronary artery disease.

The patient was monitored in the ICU for eight days following surgery. Chest pain resolved, and troponin, CK, and CK-MB levels declined, reaching a nadir of 0.2 for troponin, 204 for CK, and 19 for CK-MB on the eighth day of hospitalization, after which they remained stable. The patient also became hemodynamically stable.

## Discussion

Perioperative myocardial injury has an estimated incidence of approximately 12-24% after major non-cardiac procedures, and it is higher following non-elective surgeries. More than 80% of patients present with neither ischemia-related symptoms nor electrocardiographic abnormalities, and the illness is missed if troponin levels are not measured [6]. Takotsubo cardiomyopathy affects approximately 2 to 9 per 100,000 individuals annually in the general population [7]. About 2% of those initially suspected of having acute coronary syndrome are ultimately diagnosed with Takotsubo cardiomyopathy [8]. While in the perioperative setting, the incidence is higher, with estimates suggesting up to 1 in 6,700 patients may develop Takotsubo cardiomyopathy following non-cardiac surgery [7].

The underlying mechanisms of Takotsubo cardiomyopathy, which is a subtype of acute heart failure, remain not fully understood. Nonetheless, substantial advances have been achieved over the past decade in clarifying its pathophysiology. In most patients, clinical signs of intense sympathetic activation are present at the onset, and many describe experiencing a sudden surge of adrenaline in response to a triggering stressor [5].

Many factors, such as emotional or psychological stress, infection, surgery, medications, and exacerbation of chronic illness, can activate the sympathetic nervous system. More recently, Takotsubo cardiomyopathy has been associated with antineoplastic medications [5,9]. More than 90% of patients with Takotsubo cardiomyopathy are postmenopausal women, suggesting that estrogen deficiency may correlate with an increased risk of Takotsubo cardiomyopathy [2].

The exact mechanism for the development of Takotsubo cardiomyopathy following surgeries is unclear; however, we believe that the catecholamine surge following massive stress on the body resulted in upregulation of the beta-adrenergic receptors, causing the regional myocardial dysfunction.

Takotsubo cardiomyopathy is diagnosed using the modified Mayo Clinic criteria, which include the following: (1) transient dyskinesia, hypokinesis, or akinesis of the left ventricular mid-segments with or without apical involvement, and right ventricular involvement may be present; (2) absence of coronary artery disease on coronary angiography; (3) evidence of T-wave inversion or ST-segment elevation on the electrocardiogram; (4) a slight increase in troponin and CK levels; and (5) a lack of myocarditis or pheochromocytoma [3,4].

Our patient presented with chest pain and electrocardiographic findings concerning an anterior ST-segment myocardial infarction after major surgery. A coronary angiogram was done to rule out coronary artery disease, and Takotsubo cardiomyopathy was confirmed.

Once thought to be a benign illness, Takotsubo cardiomyopathy is now acknowledged as a serious clinical problem. Its mortality and morbidity rates are similar to those of other acute coronary syndromes and acute myocardial infarctions. Following the initial episode, many patients experience ongoing health issues, including reduced exercise tolerance, exertional dyspnea, and persistent metabolic disturbances that may last for several months. The left ventricular dysfunction that was initially believed to resolve within weeks or months, based on conventional transthoracic echocardiography, has been shown by more advanced imaging modalities, such as strain echocardiography, to not always result in full recovery of myocardial function [10].

Treatment is mainly supportive and continues until the spontaneous return of left ventricular function. Patients with mild cases may not need any intervention. Those with severe cases may require medical therapy such as angiotensin-converting enzyme (ACE) inhibitors, angiotensin II receptor blockers (ARBs), and beta-blockers [8,10].

## Conclusions

Takotsubo cardiomyopathy remains a diagnostic challenge. It should be included in the differential diagnosis for patients presenting with symptoms resembling an acute coronary syndrome. Nonetheless, immediate coronary angiography is essential in all such cases to definitively rule out an acute coronary event. We presented a case of Takotsubo cardiomyopathy after an emergent midline exploratory laparotomy. We recommend heightened clinical awareness of this potential diagnosis and encourage further research to determine the actual prevalence of this complication in patients undergoing major surgery.
